# Review Article: Contemporary Transcatheter Heart Valves for TAVI in Bicuspid Aortic Anatomy

**DOI:** 10.3390/jcm14082838

**Published:** 2025-04-20

**Authors:** Chrysavgi Simopoulou, Omar Oliva, Vincenzo Cesario, Nicolas Dumonteil, Didier Tchetche, Chiara De Biase

**Affiliations:** Groupe Cardio-Vasculaire Interventionnel, Clinique Pasteur, 45, Avenue de Lombez, 31000 Toulouse, France; chryssasimopoulou91@gmail.com (C.S.); omaroliva93@gmail.com (O.O.); vicesario91@gmail.com (V.C.); ndumonteil@clinique-pasteur.com (N.D.); dtchetche@clinique-pasteur.com (D.T.)

**Keywords:** bicuspid aortic valve (BAV), transcatheter aortic valve implantation (TAVI), balloon-expandable valve (BEV), self-expanding valve (SEV), paravalvular leak (PVL), permanent pacemaker implantation (PPI)

## Abstract

Bicuspid aortic valve (BAV) is the most common congenital heart disease, affecting 0.5–2% of the population and often leading to early aortic valve degeneration. While surgical aortic valve replacement (SAVR) remains the gold standard for treating severe bicuspid aortic stenosis (AS), transcatheter aortic valve implantation (TAVI) is emerging as a viable alternative in selected BAV anatomies. Initial experiences with first-generation transcatheter heart valves (THVs) showed the feasibility of this technique, but were associated with lower device success rates and higher complications, such as paravalvular leak (PVL) and pacemaker implantation. Advancements in second- and third- generation THVs, together with better pre-procedural imaging assessment and growing operator experience, have significantly enhanced TAVI outcomes in BAV patients, with results now comparable to those seen in tricuspid aortic valves (TAVs). Proper patient selection, pre-procedural sizing, and device implantation are key to improving TAVI success in BAV. Recent registry data on contemporary THV platforms demonstrate improved procedural success, hemodynamic performance, and the safety of TAVI in BAV. However, higher rates of PVL, pacemaker implantation, and strokes remain concerns. Ongoing advancements in THV design and procedural techniques will further enhance outcomes for this challenging population. Up to the present, there are no dedicated THVs for BAV, but the latest-generation THVs offer promising results.

## 1. Introduction

**Figure 1:**
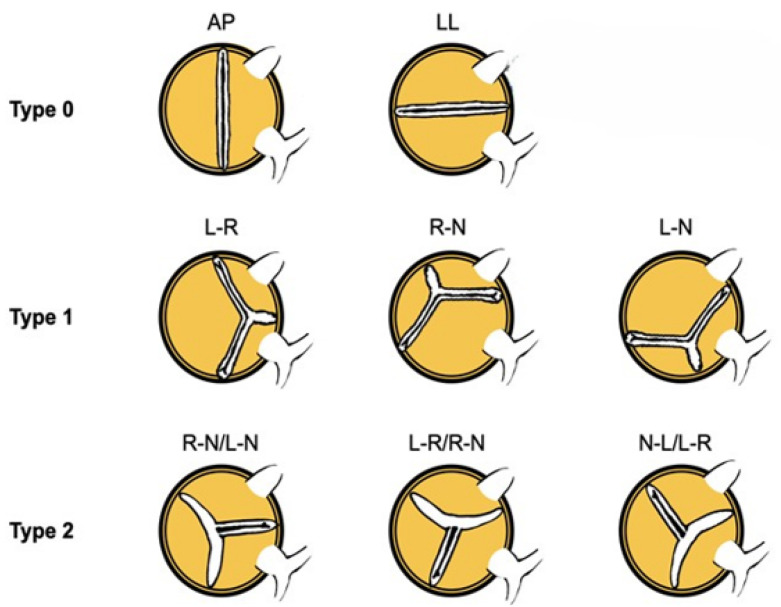
Morphological classification of the various types of bicuspid aortic valve anatomy.

Bicuspid aortic valve (BAV) is the most frequent congenital heart disease affecting 0.5–2% of the general population, with male predominance [1]. It is an anatomically complex disease [2,3], predisposing sufferers to an accelerated degeneration of the aortic valve, resulting in early disease in young patients [4]. Various classifications have been suggested to describe BAV anatomy, with the Sievers being the most widely adopted (Figure 1). Therefore, European and American [5,6] guidelines recommend surgical aortic valve replacement (SAVR) as the gold standard treatment for patients with severe aortic stenosis and diseased BAV. However, transcatheter aortic valve implantation (TAVI) in this anatomy is a valuable option in specific BAV anatomies, and is under investigation in many registries, but no data from randomized clinical trials are available. Early experience in selected BAV patients with aortic stenosis (AS) undergoing TAVI with first-generation transcatheter heart valves (THV) showed that TAVI was a safe and feasible option for this population with similar mortality when compared to TAVI in patients with tricuspid aortic valve (TAV) stenosis. However, it was associated with lower device success rates and higher rates of procedural complications, such as significant paravalvular leak (PVL), permanent pacemaker implantation (PPI), the need for a second valve and aortic root injuries [7,8,9]. New generation devices, together with the accurate pre-procedural sizing offered by multi-sliced computed tomography (MSCT) and the increasing experience of TAVI operators, have improved TAVI results in BAV, making them comparable with the outcomes of TAVI in tricuspid anatomies [10,11,12]. Yet, the proper understanding of BAV anatomy and the developments in the THV design remain crucial to further improve these results, since no dedicated devices are available for TAVI in BAV. This review aims to collect the latest available evidence of contemporary THV in BAV anatomy.

## 2. Comparison of Different TAVI Platforms in BAV

The safety and efficacy of TAVI in BAV was initially assessed in small case series using first-generation THV prostheses [13] followed by larger series treated with second-generation balloon- and self-expandable valves [7,14]. Significant improvements in the design and delivery system of third-generation valves, along with the widespread use of CT for aortic root and annulus sizing, have led to better procedural success rates of TAVI in BAV [15]. The studies comparing new generation balloon-expandable and self-expanding THVs are being summarized in Table 1.

In the international BEAT registry [16], the most commonly used balloon-expandable Sapien 3 and self-expanding Evolut R/PRO THV were compared. Both platforms showed high device success rates according to VARC-2 criteria, without significant difference between the two groups (Sapien 3 = 85.7% versus Evolut R/Pro = 84.4%; *p* = 0.821) and with comparable rates of all-cause and cardiovascular mortality at 30-day and at 1-year follow-up. Additionally, SEV THV had better hemodynamics, with lower mean gradients and higher effective orifice areas (EOAs) at follow-up. Nonetheless, in the propensity score-matched cohort, these platforms presented higher rates of moderate-severe PVL, both at discharge (10.4% versus 0.0%, *p* = 0.004) and at 1-year follow-up (9.3% versus 0.0%; *p* = 0.043), probably related to the lower radial force of the Evolut platforms and to the increased calcifications of the BAV anatomy, resulting in incomplete and elliptical THV expansion. This hypothesis was also strengthened by the more frequent pre- and post-dilatations required to achieve an optimal implantation in the SEV group (pre-dilatation: 57.3% versus 37.9%; postdilatation: 42.7% versus 14.3%; *p* < 0.001 for both). No differences were observed regarding the rates of permanent pacemaker implantation (PPI) between the two platforms, either in the overall or in the propensity score-matched cohorts. Regarding the BEV THV analysis in this paper, a trend towards higher rates of annular rupture was reported, even if it was not statistically significant. These results on SEV and BEV platforms were also confirmed in a restricted subgroup analysis of new-generation THVs, coming from a meta-analysis of eight observational studies [17]: new-generations SEVs were associated with significantly higher risk of moderate-severe PVL, even without an impact on mortality.

Another single-center registry, comparing 106 consecutive BAV patients undergoing TAVI with Evolut R/PRO or Sapien 3/3 Ultra, showed a VARC-3 device success rate of 86.8% in SEV vs. 80.9% in BEV (*p* = 0.433), with a postdilatation rate of 52.6% in the Evolut R/PRO group vs. 23.5% for Sapien 3/3 Ultra (*p* = 0.002) [18]. No significant difference in stroke/transient ischemic attack or annular rupture at 30-days follow-up were encountered. Even in this paper, a hemodynamic profile resulted better for SEV, with significantly lower gradients and larger EOAs. However, this registry showed comparable results in terms of PVL between the two platforms, with none of the patients exceeding ‘mild to moderate’ regurgitation.

Later on, the AD-HOC registry [19], an observational, retrospective, multicenter registry, enrolled patients with Sievers type 1 BAV stenosis with tapered configuration undergoing TAVI with current-generation BEVs and SEVs. The patients were analyzed with propensity score matching resulting in 301 pairs. During hospitalization, VARC-3 technical success rates were comparable between the two groups (BEVs 95.7% vs. SEVs 94.0%, *p* = 0.421). Cerebral protection devices as well as pre- and post- dilatations were more frequently applied in the SEV group. Annulus rupture occurred in two patients treated with a BEV, and the need for a second valve was numerically lower in this group (BEVs 0.3% vs. SEVs 2.0%, *p* = 0.258). Pre-discharge echocardiographic findings showed lower mean gradients (11 vs. 8 mm Hg; *p* < 0.001) and higher EOAs (1.7 vs. 2.1 cm^2^; *p* < 0.001) in the SEV group, but patients treated with SEVs presented higher rates of new PPI and moderate or greater PVL, but a lower incidence of severe patient–prosthesis mismatch (PPM) (BEVs 5.4% vs. SEVs 1.7%, *p* = 0.045). All these outcomes resulted in an overall lower rate of SEV device success and early safety. At the longer median follow-up of 1.3 years, both groups presented similar clinical efficacy in terms of mortality, neurological events and rehospitalizations for heart failure.

More recently, the TRITON study [20], a multicenter registry including 360 patients with severe BAV stenosis treated with latest self-expanding Evolut PRO+ and balloon-expandable Myval and Sapien 3 Ultra, showed an excellent safety profile of these latest generation THVs. No cases of peri-procedural death, aortic dissection, annulus rupture or coronary artery occlusion occurred in the TRITON population, and better results than those reported with previous generations THVs were reported.

In the matched cohort, Myval displayed better device success at 30 days than Sapien 3 Ultra (100% vs. 87.5%; *p* = 0.002) and the Evolut PRO+ (81.3%; *p* < 0.001). This result was mainly due to the lower residual gradients of the Myval THV and to its lower rates of more than moderate PVL. The early safety endpoint was also better with Myval (Myval 85% vs. Sapien 3 Ultra 70%; *p* = 0.031 vs. Evolut PRO+ 67.5%; *p* = 0.022). No significant differences in PPI rates among the devices were reported, whilst postdilatation was more frequent in the Evolut PRO+ group.

All these registries reported 1 year follow-up as the longest available, but longer-term data are scarce and there are concerns over the impact of the unfavorable BAV anatomy on THV hemodynamics and longevity. In this context, there is only one registry reporting the 3-years follow-up of TAVI in BAV [21]. Data were retrospectively collected from 150 consecutive patients who underwent TAVI before 2020 with various generation SEVs (CoreValve, Evolut R and Evolut PRO) and Sapien 3 BEV. In this single-center registry, favorable short and long-term clinical outcomes at 3-years follow-up were observed, including all-cause mortality, cardiovascular mortality, stroke and PPI, without statistically significant difference between the two THV groups. The VARC-3 composite endpoint evaluated in-hospital, at 30-days, 1-year and 3-years follow-up were similar between the two platform. In the SEV group, THV oversizing (SEV 1.15 ± 0.08 vs. BEV 1 ± 0.06, *p* < 0.001), pre- dilatation (SEV 59% vs. BEV 23.9%, *p* < 0.001) and postdilatation (SEV 49.4% vs. BEV 16.4%, *p* < 0.001) were more frequently adopted. At the echocardiographic follow-up, patients treated with SEV presented significantly lower residual gradients at 30 days and 1 year, with a persisting trend up to 3 years, but higher rates of moderate PVL. The limitation of this registry is the comparison of different generation SEV platforms with a first-generation BEV device, thus the results should be considered with caution.

To sum up, the comparison of SEV and BEV THVs showed, in the majority of these reports, better hemodynamic for the supra-annular SEV vs. BEV, but higher rates of pre- and post- dilatation and long-term moderate PVL, while BEV THV was more associated with the risk of annular rupture. Later in this review, available data for each platform type are reported.

**Table 1:** Summary of studies comparing new generation balloon vs. self-expanding valves in bicuspid stenotic aortic valves.

	Mangieri et al. [16] (2020)		Deutsch et al. [18] (2023)		Buono et al. [19] (2024)		Amat-Santos et al. [20] (2023)		Boiago et al. [21] (2024)		
	BEV	SEV	BEV	SEV	BEV	SEV	Sapien3 Ultra	MyVal	Evolut PRO+	BEV	SEV
N patients	77	77	68	38	301	301	80	80	80	67	83
Device success (%)	85.7	84.4	80.9	86.8	88.1	82.1	87.5	100	81.3	78.5	78.0
Pre-dilatation (%)	23.4	57.3	57.4	47.4	49.8	86.4	44.2	70.1	79.2	23.9	59.0
Post-dilatation (%)	14.3	42.7	23.5	52.6	14.6	51.2	12.8	15.4	43.6	16.4	49.4
Annular rupture (%)	2.6	0.0	0.0	2.6	0.7	0.0	0.0	0.0	0.0	3.0	0.0
Mean gradient (mmHg)	11.5 ± 4.3	8.5 ± 4.2	11.9 ± 4.5	9.2 ± 3.0	11.0	8.0	12.9 ± 4.3	8.5 ± 3.6	9.6 ± 5.2	10.7 ± 3.2	8.8 ± 3.8
EOA (cm^2^)	1.8 ± 0.2	2.1 ± 0.6	1.7 ± 0.4	1.8 ± 0.4	1.8	2.2	1.4 ± 0.1	1.8 ± 0.3	1.8 ± 0.4	-	-
≥Moderate PVL (%)	0.0	9.3	0.0	0.0	1.7	8.8	0.0	1.9	13.0	0.0	10.0
All-cause mortality (%)	5.5	4.4	0.0	2.6	10.8	14.5	2.5	0.0	3.8	28.0	28.1
Stroke/TIA (%)	0.0	1.5	2.9	2.6	4.5	5.1	7.5	1.3	3.8	6.0	12.5
PPI rate (%)	17.1	14.3	11.8	23.7	9.1	18.2	12.5	10	18.8	24.0	28.1
Follow-up duration	1 year		30 days		1.3 years		30 days		3 years		

N: number, EOA: Effective Orifice Area, TIA: Transient Ischemic attack, PPI: Permanent Pacemaker Implantation.

## 3. Evidence of Balloon Expandable THV in BAV

**Figure 2:**
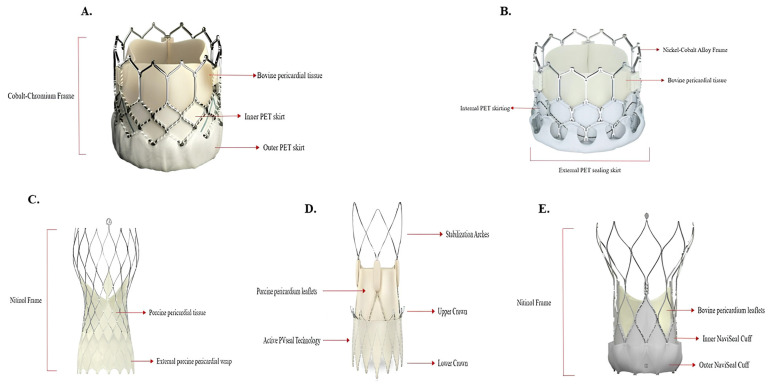
Design and structural components of the different Transcatheter Heart Valve platforms. (**A**) Sapien 3, Edwards Lifesciences; (**B**) MyVal, Meril Life Sciences; (**C**) Evolut PRO, Medtronic; (**D**) Acurate Neo2, Boston Scientific; (**E**) Navitor system, Abbott. PET: Polyethylene Terephthalate.

The majority of data on BEV platforms in BAV derive from the widely used Edwards Sapien THV. Recent papers report the outcomes of the new Sapien 3 for TAVI in BAV, underlying the novelty of the design with better outer sealing skirts and improved delivery (Figure 2A), and show increased device success when compared to older generations [15] (Table 2). In a retrospective, multicentre registry of 51 patients with BAV who underwent TAVI with Sapien 3, the device success rate was 98%, with no cases of clinically significant PVL [22]. Indeed, an oversizing of more than 10% of the annular area was related to a very low rate of PVL, without any annular rupture, and the need for post- dilatation occurred in only four cases because of significant residual intraprocedural PVL. On the other hand, the rate of PPI was higher (23.5%) than the one generally reported in patients with tricuspid anatomies treated with BEV [23].

Further data derive from a comparison between BAV and TAV treated by Sapien 3 THV [24]. The study compared 2691 BAV anatomies with severe AS with TAV-AS patients after appropriate propensity score-matching. No significant differences in 30-day and 1-year mortality between the two groups were observed, but the bicuspid cohort presented significantly higher rates of stroke (2.5% vs. 1.6%; *p* = 0.02), PPI (9.1% vs. 7.5%; *p* = 0.03) and conversion to open chest surgery (0.9% versus 0.4%, *p* = 0.03) at 30-days follow-up. Valve hemodynamics were comparable between the two cohorts, with a greater proportion of patients in the bicuspid population presenting moderate-severe PVL at discharge, together with an increased mean gradient > 10 mmHg at 30 days. These differences disappeared in longer follow-up data collection.

Later on, the prospective PARTNER 3 bicuspid registry [25] evaluated the efficacy of Sapien 3 in low surgical risk patients with BAV and severe AS. In this study, 148 BAV were compared to a propensity score-matched population of TAV undergoing TAVI with Sapien 3 platform, showing comparable rates of death, stroke, rehospitalization and PPI at 30-days and 1-year follow-up. In addition, echocardiographic findings on valve hemodynamics and PVL were also similar between the two populations. Nevertheless, this registry has an important limitation, since patients with severe raphe or left ventricular outflow tract calcifications were excluded, making it difficult to validate these findings for more complex BAV anatomies undergoing TAVI with this balloon-expandable platform.

Looking at a different balloon-expandable THV, data on the MyVal platform (Figure 2B) in BAV were recently published. The authors reported the 1-year clinical and echocardiographic outcomes of 62 patients with BAV undergoing TAVI with this THV [26]. In this multicenter registry, the rate of all-cause mortality was 11.3%, with only three reported cases of cardiovascular mortality (4.8%) and six of cardiovascular-related rehospitalization (10.6%). All-stroke and PPI rates were low, 3.2% and 8.3%, respectively, at 1-year follow-up. As for device-related complications, severe bioprosthetic valve dysfunction (BVD) and valve thrombosis were detected in one patient each, whilst a moderate hemodynamic deterioration was reported in three patients. Previous data had already shown the safety and efficacy of MyVal in BAV in terms of device success, all-cause mortality, stroke and residual PVL at 30 days [27], but the longer follow-up of this study revealed the stable hemodynamic performance of the device over the 1-year follow-up, with a mean gradient of 10 mmHg, an EOA of 1.7 cm^2^ and a moderate PVL in only 2% of the cohort.

The safety and efficacy of MyVal in patients with BAV were also confirmed in another single-center study comparing 52 pairs of BAV and TAV patients [28]. In this registry, there were no significant differences between the two groups regarding device success, all-cause and cardiovascular mortality, stroke rate, major vascular complications and PPI rate, both at discharge and at 30-day follow-up. However, the PPI rate was high in both groups (BAV 34% vs. TAV 24%, *p* = 0.274), with no significant differences in pre-procedural characteristics despite a tendency towards higher aortic valve calcium scores in BAV patients. Finally, the hemodynamic performance both at discharge and at 30 days did not differ between the two groups, and no moderate or above PVL was observed in the bicuspid patients.

Overall, the available BEV platforms showed good device success in BAV population, despite the need for post-dilatation in order to avoid major PVL for the Sapien 3 THV, and the high PPI rate for the MyVal, probably related to the oversizing and the radial force of this THV.

**Table 2:** Summary of studies evaluating new generation balloon-expandable valves in bicuspid anatomy.

	Perlman et al. [22] (2016)	Makkar et al. [24] (2019)	Williams et al. [25] (2022)	Elkoumy et al. [26] (2023)	Magyari et al. [28] (2024)
THV platform	Sapien 3	Sapien 3	Sapien 3	MyVal	MyVal
N patients	51	2691	148	62	52
Device success (%)	98.0	96.5	100.0	100.0 [27]	100.0
Mean gradient (mmHg)	11.2 ± 4.7	13.1 ± 8.1	-	10 (8–16.5)	9.7 ± 3.5
EOA (cm^2^)	1.68 ± 0.32	-	-	1.7 (1.4–1.9)	-
≥Moderate PVL (%)	0.0	3.2	3.1	2.0	0.0
All-cause mortality (%)	3.9	10.5	0.7	11.3	0.0
Stroke/TIA (%)	1.9	3.4	2.1	3.2	1.9
New PPI rate (%)	23.5	10.0	6.8	8.3	34.0
Follow-up duration	30 days	1 year	1 year	1 year	30 days

## 4. Evidence of Self-Expanding THV in BAV

Data on SEV platform in BAV derive largely from the most frequently Medtronic Evolut R and Evolut PRO THVs (Table 3). The features of these platforms consist in the recapturable capability and the optimal pericardial outer wrap in the PRO version (Figure 2C).

Data from the Society of Thoracic Surgeon/American College of Cardiology Transcatheter Valve Therapy (STS/ACC TVT) registry [29] showed no significant difference in all-cause mortality at 30 days or 1 year between the 929 pairs of BAV and TAV patients undergoing TAVI with Evolut R or Evolut PRO (30-day mortality 2.6% vs. 1.7%; *p* = 0.18, 1-year mortality 10.4% vs. 12.1%; *p* = 0.63). Also, there was no significant difference in stroke rate both at 30 days (3.4% vs. 2.7%; *p* = 0.41) and at 1 year (3.9% vs. 4.4%; *p* = 0.93). However, a small but increased number of patients in the bicuspid group required aortic valve reintervention in comparison with the tricuspid group at 30 days (0.8% vs. 0.1%; *p* = 0.03) and at 1 year (1.7% vs. 0.3%; *p* = 0.01). Nevertheless, the hemodynamics of these THVs resulted in excellent outcomes in both groups, with a mean transvalvular gradient at 30 days and at 1 year less than 10 mmHg. Even if the immediate post-procedural gradients were slightly higher in the BAV group (9.7 ± 5.2 mm Hg vs. 9.0 ± 5.0 mm Hg; *p* = 0.002), this difference disappeared at 1 year control (9.4 ± 5.2 mm Hg vs. 8.9 ± 5.1 mm Hg; *p* = 0.22). In addition, moderate or severe PVL was more frequent in the BAV group at 30 days (5.6% vs. 2.1%; *p* < 0.001), partially justifying the higher incidence of reinterventions in this group. However, in this study, less than one-third of the patients in each group underwent TAVI with the Evolut PRO platform, and when comparing the two valve types, the incidence of greater than mild PVL was lower in patients treated with the Evolut PRO, both in bicuspid and tricuspid patients at 30 days (2.2% vs. 1.5%; *p* = 0.71).

More data were collected from the Evolut Low Risk TAVI Bicuspid Study [30], a prospective, single-arm study, enrolling 150 low-risk patients with BAV and AS who underwent TAVI with Evolut R or PRO THV. In this study, the device success rate was 95.3% and the initial rate of all-cause mortality or stroke at 30 days was low at 1.3%. A second valve was implanted in five patients (3.3%) and a new permanent pacemaker in twenty-two (15.1%) at 30-days follow-up. This last rate was slightly lower than the one observed in low-risk TAV patients (17.4%) [31]. Regarding the valve performance, at 30 days there were no patients with more than mild PVL, the mean gradient was 7.6 (±3.7) mmHg and the EOA was 2.3 (±0.7) cm^2^. Recently, the 3-year outcomes from this study were also published, showing a rate of all-cause mortality or disabling stroke of 4.1%, and a new PPI rate of 19.4% occurring during the first year [32]. At 3-year follow-up, the mean gradient remained low at 9.1 ± 5.8 mmHg, with no change in the EOA and no case of moderate or severe PVL. Moreover, only two patients required surgical aortic valve reintervention.

The comparison of 145 pairs with low risk BAV and TAV anatomies showed no significant difference in all-cause mortality or disabling stroke (1.4% vs. 2.8%, *p* = 0.413) and PPI (16.6% vs. 17.9%, *p* = 0.741) rate at 1-year follow-up [33]. Mean gradient (8.7 ± 3.9 mmHg vs. 8.5 ± 3.1 mmHg, *p* = 0.754) and EOA (2.2 ± 0.7 cm^2^ vs. 2.3 ± 0.6 cm^2^, *p* = 0.677) were also similar between the two groups, but the incidence of mild or greater PVL was higher in patients with tricuspid anatomy (21.3% vs. 42.6%, *p* < 0.001).

In the international, prospective Bivolut X registry [10], 149 BAV patients were treated with an Evolut R or PRO 34 mm valve using two different sizing strategies; the first one according to the annulus size and the second one using a combination of annular and supra-annular dimensions. In this registry, at 30 days, the device success rate was 91.3% and the cardiac death rate was 2.6%, increasing to 11.0% after 1 year. The mean gradient was low at 7.2 (5.4–9.5) mmHg and the EOA was large at 2.1 (1.8–2.6) cm^2^, with no patients having severe PVL. Only two patients presented severe patient–prosthesis mismatch (PPM) at 30 days. Clinical and echocardiography results did not differ between the two sizing strategies both at 30-days and 1-year follow-up. In this registry, the stroke rate was 4.6%, and it was attributed to the high calcium burden of BAV and to the high incidence of pre- and post- dilatations required in the majority of patients. Additionally, the new PPI rate was 19.5% at 30 days and 25. 6% at 1 year, which is higher than the one reported in TAV patients [31], but similar to the one observed in the aforementioned Evolut Low Risk TAVI Bicuspid Study [30]. It is worth mentioning that in this study, the investigators did not systematically use the cusp overlap technique in the time of the enrollment, which could partially explain the high PPI percentage.

Recently, new evidence on a different self-expanding platform were published in the Neo2 BAV registry [34] regarding the use of the ACURATE neo2 valve (Figure 2D) in bicuspid anatomies (Table 3). In this retrospective, multicenter registry, 181 BAV patients underwent TAVI with this platform. At 30 days, the device success rate was 90.6% and the early safety rate was 82.3%, the cardiovascular death and stroke rates were low at 2.2% and 1.6%, respectively, while the need for a second valve occurred in 2.2% of all cases. The hemodynamic results at 30 days were also encouraging, with a mean gradient of 6.5 (4.6–9.0) mmHg and a median EOA of 2.0 (1.7–2.5) cm^2^. One patient presented severe PPM, and there were no cases of severe PVL but moderate PVL was noted in 4.3% of patients. These results were comparable with those reported in the aforementioned registries using the Evolut R and PRO platforms [10,30]. Finally, the incidence of new PPI at 30-days follow-up was 6.5%, the lowest reported in BAV anatomies after TAVI.

All considered, SEV platforms showed enthusiastic results, with low gradients, large EOAs and low incidence of PVL, despite the need for PPI in the Evolut more than in the Acurate series, probably because of the radial force of the first and for the need of pre- and post-dilatation, together with an implant in a ‘three cusps’ view.

Up to now, no data have been made available for the third available SEV Navitor THV by Abbot in BAV (Figure 2E). This new-generation of the Portico self-expanding THV, presenting a more flexible delivery system, large cells and an active sealing, showed very enthusiastic results in tricuspid patients, with mean gradients of 7.5 mmHg and EOAs of 1.9 cm^2^ at 1-year follow-up [35]. Despite the use of this THV through lower-risk populations, no published data are currently available for the treatment of bicuspid anatomies. However, recent data from an ongoing multicenter registry [36] with a total of 47 patients with BAV treated with Navitor have shown a high procedural device success rate of 91%, with an early safety rate of approximately 70%; these results are encouraging and probably this platform could provide an effective and safe option for BAV patients undergoing TAVI.

**Table 3:** Summary of studies evaluating the new generation self-expanding valves in bicuspid anatomy.

	Forrest et al. [29] (2020)	Zahr et al. [32] (2024)	Thetche et al. [10] (2023)	Ruck et al. [34] (2024)
THV platform	Evolut R/PRO	Evolut R/PRO	Evolut PRO/R34	Acurate Neo2
N patients	929	150	149	181
Device success (%)	96.5	95.3 [30]	91.3	90.6
Mean gradient (mmHg)	9.4 ± 5.2	9.1 ± 5.8	8.1 (6.2–11.1)	6.5 (4.6–9.0)
EOA (cm^2^)	-	2.2 ± 0.7	2.1 (1.8–2.5)	2.0 (1.7–2.5)
≥Moderate PVL (%)	4.7	0.0	1.6	4.3
All-cause mortality (%)	10.4	2.8	10.0	2.2
Stroke/TIA (%)	3.9	6.9	7.1	1.6
PPI rate (%)	16.4	19.4	25.6	6.5
Follow-up duration	1 year	3 years	1 year	30 days

## 5. Limitations and Future Directions

Several limitations remain in the current body of evidence. Most studies are observational, with limited long-term data and significant heterogeneity in patient selection, BAV morphologies, imaging criteria, and valve types. Critically, no randomized controlled trials have directly compared BEV and SEV platforms in this population. Moreover, most registries exclude patients with complex BAV anatomy, such as those with extensive raphe calcification or asymmetric leaflet fusion, limiting generalizability.

To optimize outcomes in BAV patients undergoing TAVI, future research should focus on randomized trials with head-to-head comparisons of BEV vs. SEV platforms; the development of BAV-specific THVs designed to accommodate the unique anatomical challenges of bicuspid valves; long-term follow-up studies assessing durability, valve degeneration, and clinical outcomes beyond 1–3 years; and standardized imaging and sizing protocols to improve pre-procedural planning and reduce complications like PVL or annular rupture.

## 6. Conclusions

As TAVI has expanded as an effective treatment even in young and low risk patients, the proportion of patients with BAV anatomy undergoing TAVI is progressively increasing. No dedicated device is available for this anatomy, but evidence of available platforms, originally designed for tricuspid aortic valves therapy, seem to have good results even in BAV [37,38,39]. Appropriate patient selection and THV sizing are cornerstones for the success of the procedure, especially in the heterogeneous field of bicuspid anatomies. Improvements in current available devices and operator practice, as well as multimodality imaging analysis, could make this procedure even more effective for AS treatment in BAV. Additional evidence from different platforms in bicuspid or new dedicated BAV devices need to validate this treatment.

## Data Availability

No new data were created or analyzed in this study. Data sharing is not applicable to this article.

## Abbreviations

BAV	Bicuspid Aortic Valve
AS	Aortic Stenosis
TAV	Tricuspid Aortic Valve
EOA	Effective Orifice Area
VARC	Valve Academic Research Consortium
PPM	Patient–Prosthesis Mismatch
BVD	Bioprosthetic Valve Dysfunction
MSCT	Multi-Sliced Computed Tomography
THV	Transcatheter Heart Valve
BEV	Balloon-Expandable Valve
SEV	Self-Expanding Valve
PVL	Paravalvular Leak
PPI	Permanent Pacemaker Implantation
SAVR	Surgical Aortic Valve Replacement
TAVI	Transcatheter Aortic Valve Implantation
